# From trauma to transmission: exploring the intersection of adversity, substance use, and HIV risk in women’s life histories

**DOI:** 10.1186/s12939-023-01994-4

**Published:** 2023-09-01

**Authors:** Nora S. West, Frank Kussaga, Alex Rittenhouse, Brenice Duroseau, Deja Knight, Jessie Mbwambo, Samuel Likindikoki, Haneefa T. Saleem

**Affiliations:** 1 Division of Pulmonary and Critical Care Medicine, University of California San Francisco, San Francisco, CA USA; 2 Internal/Preventive Medicine, Griffin Hospital, Derby, CT USA; 3 Department of Environmental Health, Bloomberg School of Public Health, Johns Hopkins University, Baltimore, MD USA; 4 Johns Hopkins University School of Medicine, Johns Hopkins University, Baltimore, MD USA; 5 School of Nursing, Johns Hopkins University, Baltimore, MD USA; 6 Department of International Health, Bloomberg School of Public Health, Johns Hopkins University, Baltimore, MD USA; 7 Department of Psychiatry and Mental Health, School of Medicine, Muhimbili University of Health and Allied Sciences, Dar es Salaam, Tanzania; 8 School of Public Health and Social Sciences, Muhimbili University of Health and Allied Sciences, Dar es Salaam, Tanzania

**Keywords:** HIV, Drug use, Trauma, Women’s health

## Abstract

**Background:**

At increased risk for poor health outcomes, physical and/or sexual violence, and onward transmission of HIV, women who use drugs and are living with HIV (WWUDHIV) are vulnerable and in need of services. Understanding the role of trauma across their life history may offer insights into HIV and drug use prevention and opportunities for intervention. We explored trauma and drug use among WWUDHIV in Dar es Salaam, Tanzania.

**Methods:**

We conducted in-depth interviews with 30 WWUDHIV from January-March 2019. Interviewers used semi-structured interview guides and asked questions about the life history as related to drug use. Interviews were audio recorded, transcribed, translated, coded, and life histories charted. We utilized content analysis.

**Results:**

Participants described death of family members as traumatic catalysts for drug use. Sexual partners early in their life history were often the point of introduction to drugs and source of HIV acquisition. Death of partners was present across many life histories and was a traumatic event negatively influencing life trajectories, including start of sex work for survival or to support drug use. Sex work in-turn often led to traumatic events including sexual and/or physical violence. HIV diagnosis for many participants followed the start of drug use, frequently occurred during pregnancy or severe illness and was described by most participants as a trauma. Despite this, particularly during pregnancy, HIV diagnosis was a turning point for some participant’s desire to engage in drug use treatment. Traumatic events were often cumulative and regularly described as catalysts for poor mental health that could lead to new or increased drug use for coping.

**Conclusions:**

These findings suggest trauma is common in the life history of WWUDHIV and has negative impacts on drug use and HIV vulnerability. Our life history charting highlights the cumulative and cyclical nature of trauma and drug use in this population. This study allows for better understanding of trauma, drug use, and HIV prevention, which offers opportunities for intervention among a group with limited access to services: during adolescence for orphaned youth, following the death of a child or partner, and when vulnerable women engage with the health system (HIV diagnosis, pregnancy, illness).

## Background

Drug use is a public health issue of importance given its impact on life expectancy, quality of life, and role in the acquisition and onward transmission of infectious diseases, including HIV [1–3]. In Africa, and particularly East Africa, heroin enters the region via drug trafficking routes, making it accessible and relatively inexpensive to those residing in the countries and cities that serve as hubs along trading lines [4]. Along with accessibility, multilevel social and economic drivers including lack of psychological support, exposure to violence, limited social support, and lack of economic opportunity can further contribute to heightened vulnerability to drug use for women in East African settings [5]. Overall, drug use has been on the rise in East Africa for more than two decades and is projected to continue to increase [5].

Women who use drugs (WWUD) remain vulnerable and disproportionately impacted by HIV, despite critical advances in programming and policy, highlighting a need for more targeted efforts to increase HIV testing and treatment in Africa [6]. HIV prevalence estimates among WWUD in Tanzania are considerably higher than the general population, with estimates ranging from 25 to 41% [7, 8], illuminating a crucial focal point for the provision of supportive services. Despite this need, engagement in HIV care and retention in antiretroviral therapy (ART) remain a persistent challenge among WWUD [9, 10].

HIV, trauma, and substance use are syndemic and disproportionately affect vulnerable populations, yet remain understudied and under prioritized [11]. Studies from numerous settings have shown WWUD, including heroin, are more likely to have experienced trauma, physical and/or sexual violence, and poor mental health [12, 13]. Definitions vary, but trauma can be broadly defined as experiences that cause intense physical and psychological stress reactions which may include exposure to death, serious injury, or violence, or an event that overwhelms the individual’s ability to respond [14]. Childhood or adolescent trauma is prominent in the lives of WWUD [15, 16], and trauma during childhood and adolescence is associated with poor mental health and increased likelihood of substance use/abuse [17]. Trauma can be both a cumulative factor and/or serve as a single triggering event in the context of HIV acquisition and substance use [18]. Cumulative trauma - the experience of multiple traumatic experiences throughout a person’s life - can leave a lasting imprint on one’s psychological and physical health if left unaddressed [19–21]. As a cumulative factor, the impact of multiple traumatic experiences can accumulate throughout the life course, increasing one’s vulnerability to HIV acquisition and likelihood of engaging in substance use. As a precipitating event, one traumatic experience can be the turning point that leads to the initiation or escalation of substance use and heightens the risk of HIV acquisition. Taking any type of trauma into account in the context of substance use is crucial for understanding trauma’s influence on HIV risk and management, considering its link to heightened HIV vulnerabilities; poor health outcomes due to adverse internal and external mediators; and suboptimal adherence to treatment and advancement along the HIV care continuum [22].

Much of the research on WWUDHIV comes from high-income settings, with limited evidence from women in low-income African settings like Tanzania. Women who use drugs and are living with HIV (WWUDHIV) in such settings may have unique experiences and encounter challenges due to a lack of economic independence leading to high engagement in sex work, experiences of violence and stigmatization [16, 17, 23]. Understanding when and how trauma and significant life events occur among women may be key to identifying points for intervention – both for drug use and HIV prevention among this vulnerable population.

Life histories have been used as a tool in research to understand convergent and divergent factors that relate to the phenomena of interest across the life course. This approach may be particularly useful when delving into sensitive topic areas that can be stigmatizing given that life histories allow participants to lead the narrative [24]. Because of the participant-led nature, life history approaches have been used in research exploring trauma, drug use, HIV, and sex work [24–26]. The goal of this study was to explore the role of influential life events across the life history as it relates to HIV and drug use among women, in addition to understanding factors that may mitigate the impacts of trauma among WWUDHIV in Dar es Salaam, Tanzania.

## Methods

### Study setting and population

From January and March 2019, we conducted 30 semi-structured in-depth interviews (IDIs) with WWUDHIV in Dar es Salaam, Tanzania. Participants were adult women, aged 18 and above, who reported heroin use in the past 30 days, in order to obtain a sample of adult women who were current heroin users. Data from this study were drawn from a parent study examining HIV prevention and treatment among heroin-using women and their social supporters in Dar es Salaam [10]. In the parent study, a cross-sectional survey was conducted among 200 women who reported heroin use. Participants in the survey were recruited through respondent-driven sampling via other women who reported heroin use with initial seeds identified by community outreach workers who offer services to people who use drugs (PWUD). Among the 56 women living with HIV in the survey, 30 were purposively selected to participate in qualitative interviews.

### Procedures

IDIs were conducted by trained qualitative interviewers in Swahili in a study office in Dar es Salaam. All participants provided informed consent. A semi-structured interview guide was used for interviews which covered topics related to using drugs and HIV care and treatment experiences. Interviews followed a life history approach [27], with participants asked to think back on their life and describe high, low, and turning points, along with challenges and positive and negative influences across their lives. Interviews lasted between one and two hours. All interviews were audio recorded. Interviewers completed debrief memos immediately following each interview, which were reviewed and discussed at study meetings. Completed interviews were transcribed and translated into English.

### Analysis

Content analysis: A codebook was developed by study team members, with primarily inductive codes derived from the data, and some deductive codes based on the interview guide. Coding included high, low, and turning points in participants’ lives, emotions, and related experiences including hopelessness and suicidality, loss, shame, stigma and discrimination, vulnerability, and motivations for HIV and drug use treatments. Line-by-line coding was conducted on an initial set of transcripts and used to develop a set of codes. A second cycle of coding was used to develop focused codes, which resulted in a finalized codebook that was applied to all transcripts using NVivo 12 (QSR International) for coding and data management.

Life history analysis: Participants’ life histories were captured via life history charts. Charts, based on interview transcripts, followed a timeline and focused on key events in the life of WWUDHIV of trauma and other influential time points as related to drug use and/or recovery. Charting was used as a tool to establish patterns across the life histories within the sample. Drawing upon the coding definitions established during the content analysis process, events related to the research area of inquiry were classified as point events when participants mentioned significant experiences or moments that changed or influenced their life or life course. For charting, these were further categorized as “high”, “turning”, or “low” events as they related to women’s life trajectories and their drug use or recovery journey. High point events were charted as events viewed as positive by participants, low events were those that were viewed as events with negative influences or events that detrimentally impacted women’s lives and drug use, and turning events were those that were not necessarily positive or negative, but events that were times of change. Catalyst events were events that led or dissuaded participants to use drugs. Traumatic events were those that participants described as causing significant physical and/or psychological distress. Traumatic events could be physical or psychological and were classified as traumatic based on participant descriptions of intense stress reactions. Spanning states were descriptions of ongoing factors in participants’ lives that were not necessarily tied to a single event but were part of the trajectory of their lives. These could be both tangible, like stages in the life course (e.g., adolescence or adulthood) or ongoing engagement in sex work, or emerge through descriptions in the transcripts, such as states of poor mental health and social support. One study team member, N.S.W., charted each life history based on interview transcripts. A second study team member who was part of the original data collection team and a native Swahili speaker, F.K., reviewed, clarified meaning, and verified all life history charts based on transcripts and revisited Swahili versions of the original interviews as needed to clarify meaning. Two additional study team members, A.R. and D.K., charted a random selection of ten life histories. All charts were reviewed and compared by the study team, with discrepancies in charting discussed and revised in final charts based on consensus.

As part of the analysis, events and commonalities found across the life history charts were compared to the codes and coded transcripts from the content analysis to verify patterns and themes emergent from the charting. All analytic study team members met regularly to discuss their interpretations and emergent trends and themes from the process.

## Results

**Fig. 1:**
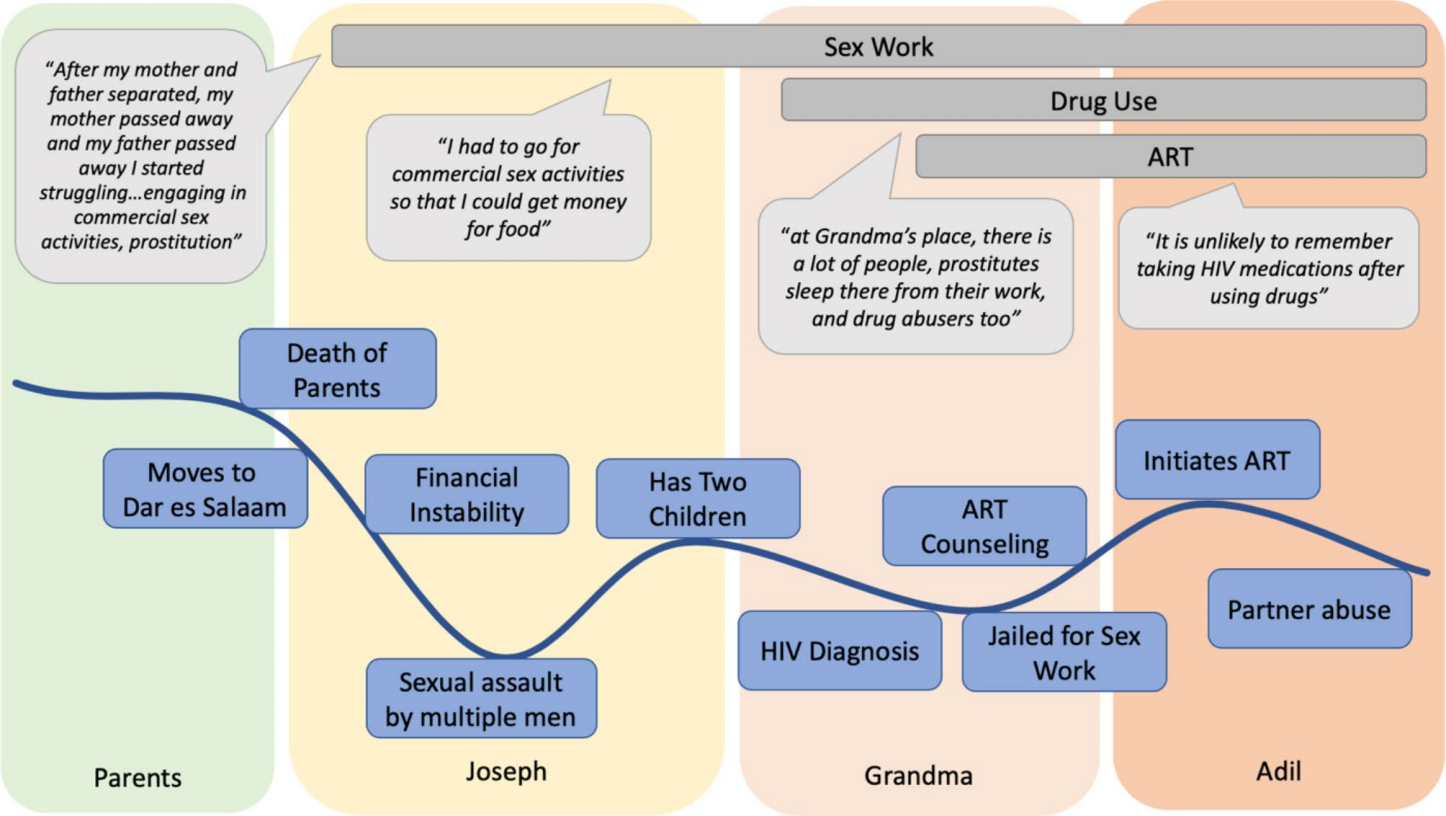
Life history chart: Amina

**Fig. 2:**
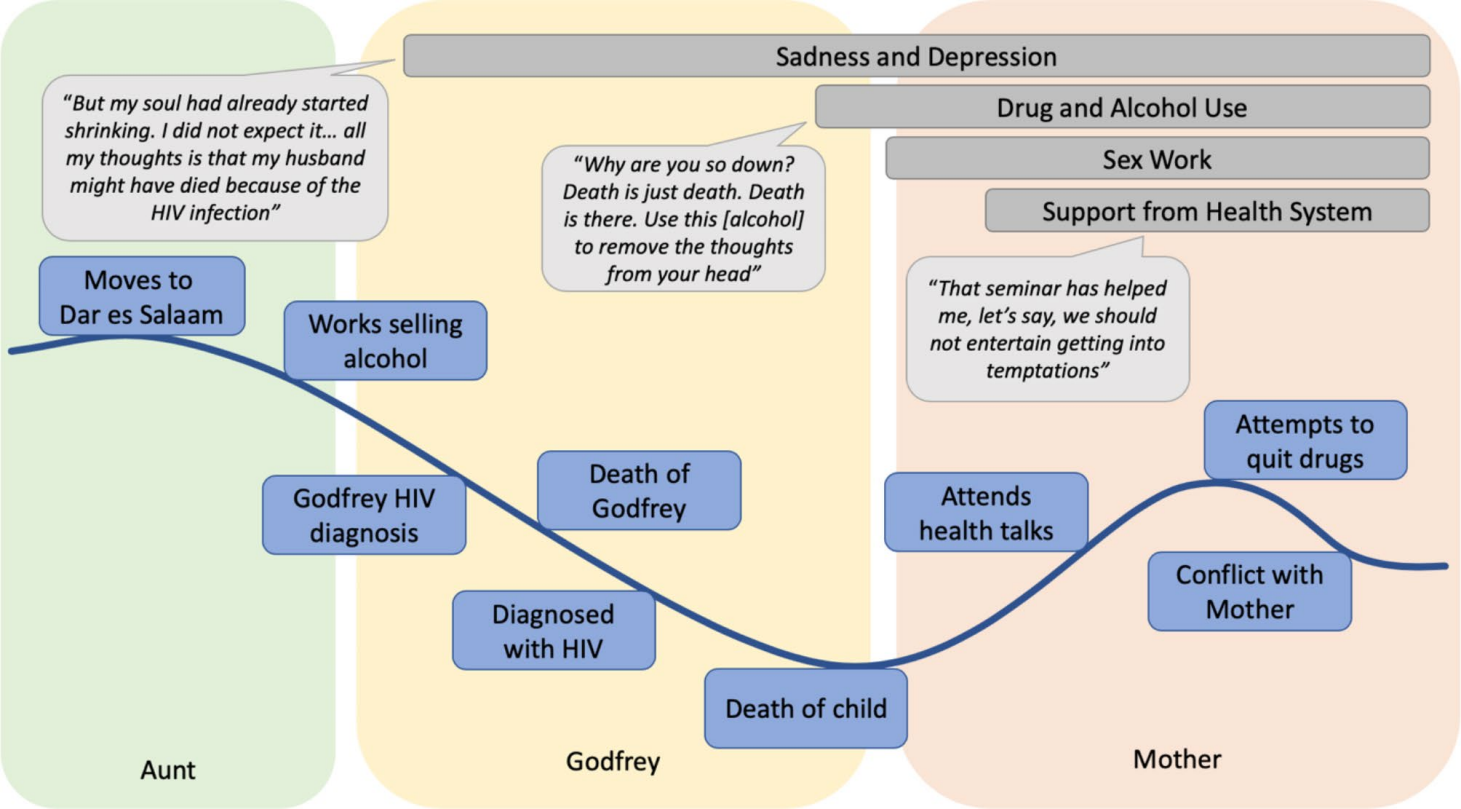
Life history chart: Neema

Findings are presented by key topic areas across the life history. Out of the WWUDHIV (n = 56) from which the qualitative sample (n = 30) was drawn, the majority (59%) were between 29 and 39 years old, had primary school or less education (88%), were married or had a partner (53%), and used heroin daily (80%). Figures 1 and 2 present the life histories and narratives of two participants (all names are pseudonyms). These life histories were chosen because of their representation of shared themes found across participant interviews and are exemplary of the life trajectories of women included in the study.

Figure 1**Narrative**: Amina described a happy life living at home with her parents in adolescence. After the separation and subsequent death of both of her parents - a turning point and catalyst for drug use - she moved to Dar es Salaam from her family home and began sex work. *“After my mother and father separated, my mother passed away and [afterward] my father passed away I started struggling… engaging in commercial sex activities, prostitution.”* In Dar es Salaam she met Joseph and they had two children. Amina described being raped by four men as a traumatic event and low point in her life that she struggled to stop thinking about and caused her sadness she “*cannot forget”*. Additionally, Amina described coercion to provide condom-less sex to sex work clients as distressing. She was diagnosed with HIV during this same period, though was unaware of the source of infection. Joseph did not want her to engage in sex work, but also did not support her financially. *“I had to go for commercial sex activities so that I could get money for food. He [Joseph] just used to stay home and wait for food, he used to eat when I cooked for my children. He was not any help.”* She described how she engaged in sex work initially to support her children. She later moved to a house with an older woman referred to as “Grandma”. While Grandma provided housing and childcare, as well as encouraged Amina to manage her HIV and take ART, the house was also home to other women selling sex and individuals would go to the house to use drugs: *“Yeah, at Grandma’s place, there is a lot of people, prostitutes sleep there from their work and drug abusers too.”* Further, Amina reported that relatives of Grandma would often disclose her HIV status to others without her permission, which made her feel stigmatized. Amina started using drugs, specifically smoking heroin, after she moved in with Grandma. Amina described the struggle to maintain ART adherence due to ongoing drug use, though she at times attended seminars or spoke with community outreach workers about ART adherence and stopping drug use. She described meeting her partner, Adil, as a turning point in her life. Adil encouraged her to quit drug use and sex work, which she did attempt though unsuccessfully. Adil did not support her financially and so she ultimately continued sex work to support her drug use. Amina described Adil as physically and verbally abusive.

Figure 2**Narrative**: Neema left her village to live with her aunt in Dar es Salaam in her adolescence, a turning point that put her on a path to drug use. The aunt told her she would help her find a job as a maid but instead had her work selling alcohol when she arrived. She met Godfrey while selling alcohol and had four children with him. *“But, fortunately, but also unfortunately, I did the chibuku [alcohol] job and got a boyfriend.”* Godfrey became ill and was diagnosed with HIV when hospitalized and subsequently died while Neema was five months pregnant with their fifth child, which placed her in a precarious financial situation and lead her to question her own HIV status. About a month after the death of her husband, Neema tested positive for HIV. Shortly after Godfrey’s death, her youngest child contracted tuberculosis and died. Neema described being treated poorly and being told she had failed by a doctor just before her child’s death. The cumulative trauma from the deaths of her husband and child were events that catalyzed future behavior, negatively impacted her mental health, and set Neema on a course toward drug use. She was selling sex at this time. She began using alcohol, and subsequently smoking marijuana and heroin after the death of Godfrey and her child to combat feelings of grief, sadness, loneliness, and depression. *“The thing that made me to use alcohol is stress because I become lonely. I meet with my girlfriend who used alcohol. When I became down, they start: ‘What is with you? Why are you being so down? Death is just death. Death is there. Use this to remove the thoughts from your head.’ So, I see it is okay. Let me just use it. I use it.”* Despite having initiated ART after being diagnosed with HIV, Neema described periods of poor health and regular secondary infections, as well as challenges with adherence due to drug use. She described support from her mother at times, with whom some of her children lived. This support, while mostly useful, ultimately culminated in conflict due to her ongoing drug use, a low point for Neema.

In this analysis, we identified patterns of vulnerability for WWUDHIV. Loss of family and abuse early in childhood were common catalyzing events that altered WWUDHIV’s life trajectories. Frequently resulting from catalyzing events, WWUDHIV described migration from their birth homes, sexual and intimate partners, and involvement in sex work as turning points that led to drug use. Physical and sexual violence and HIV acquisition were frequently described as traumatic time points in WWUDHIV’s lives. Woven throughout participant narratives were the impacts of catalyzing events, turning points, and cumulative trauma on mental health.

### Parental death and adverse childhood experiences

Traumatic and other adverse childhood experiences were catalysts for drug use for many participants and were described as low points in their lives. These events frequently involved the death of a parent, or parents, or separation from parents. When describing the loss of her mother and the mental health impact that this loss continued to have on her as an adult, one participant explained:

*Since my childhood, I was never happy because I have no mother…That is what makes me very sad because I’ve had to toil on my own while I know that if I had [my mother], the burden would have been less. A mother is different from other people and stands by you through thick and thin. -*WH04.

The death of a parent, or parents, as a child placed women in vulnerable positions, which led some of them to be mistreated or abused by relatives whose care they were placed under. One participant described her experience of being raised by an aunt following the death of both of her parents:

*I was taken to be raised by my aunt. Auntie abused me a lot. And I decided to leave her home because it reached the point where my aunt stabbed me with a knife here because of two hundred shillings, which was for buying sardines. -*WH20.

### Migration to Dar es Salaam

Migration from home villages or smaller cities to Dar es Salaam was a turning point that put many participants interviewed on a path to drug use and HIV acquisition risk. Social and economic vulnerabilities were frequently described by participants as both reasons for migrating and consequences of migrating. Reasons for migration were often linked to a parent’s or caregiver’s death and/or extreme financial hardship that pushed women to seek “a better life” in Dar es Salaam, the commercial capital of Tanzania. For some participants, migration led to the introduction and often a reliance on intimate partners or friends who in turn introduced them to drugs:

*When my sister died then I was left at home…I left home to look for a better life then I met my friend who influenced me to start using drugs. That friend used to go and steal things and when she came back, we enjoyed the drugs together.* -WH22.

The period immediately following migration to Dar es Salaam, forced some women into housing instability—“*Life was tough sleeping at the ferry*”—and job situations that led to their exploitation and, in a few cases, exposed them to violence. For example, one participant, an orphan, described migrating to Dar es Salaam with a man who found her a job at a restaurant. Not having a stable place to stay, she slept at the restaurant. One night while sleeping at the restaurant, she was sexually assaulted by multiple men. Though this was an extreme case, the broader themes of social and economic vulnerabilities associated with migration, and the traumatic events stemming from these vulnerabilities, were consistent across many participants.

### Involvement in sex work

Most participants described engaging in sex work to support their drug use:

*We went to the place called “Rambo” it’s around Morogoro road. So, when we reached there, I discovered that going to the ‘road’ means sex work. And since I needed money, there was no way out. I decided to engage myself in that work and started to go to the ‘road.’ I would get money and we would return to our camps and smoke [heroin].* -WH22.

Though most participants ultimately engaged in sex work to support drug use, some participants described beginning sex work for survival, a turning point that led to their introduction to drugs, including heroin: “*We started to go to the roadside to sell our bodies… then later I started using drugs. When I started abusing drugs, I stayed using drugs for a long time. It reached a point where no one wanted me anymore.”*-WH12.

### Intimate relationships

Sexual or intimate partners, present early in the life history for some participants or introduced soon after migration, were regularly described as the catalyst for drug use. For a number of participants, these partners were engaged following loss, hardship, or migration from villages to larger cities. One participant compared her life before and after meeting a partner: “*In the past I had good life. Then later things changed when I met and live with a man who was using drugs. When I started living with him then I entered into drugs*.” -WH30. For some women, partners were also an initial source of conflict and cause for separation from their families which could further put them on a path to drug use, isolation, loss, poor mental health, and limited support, as described by a participant:

*I entered into the relationship with him without knowing if he is a drug user, he mixes drugs in cigarettes and smoke as if he is smoking a cigarette. He tells me, “let us smoke together” and since he is my partner, I said, let me smoke to satisfy him. From there onwards I started smoking and we used to get out together. When my family discovered that I am using drugs, they separated from me and therefore I accompanied him [partner], who later God took him, he died and since then my life shook.* -WH01.

### Experiences of violence

Stories of traumatic physical and sexual violence were common across life histories, and frequently linked to selling sex to support ongoing drug use. Participants discussed how women, unlike men, had limited ways to support their drug use aside from selling sex. This made them particularly vulnerable and put them at greater risk for violence and trauma, which could, in turn, have negative impacts on mental health:

*Ehee! I have encountered such things, having sex by force. I have encountered such things. It has affected me. I have become a person who is like… that is why I don’t want to think about them [these experiences] sometimes because when I think about them I feel guilty. You know? I feel like I am someone who should not live. I am not worthy.-*WH02.

Several participants suggested trauma from sexual and intimate partner violence was a catalyst for mental distress, with women describing feelings of depression, hopelessness, worthlessness, and, in some cases, suicidality. Among those who described trauma and its impacts, the effects were enduring, spanning states in their lives. These traumatic events were often also low points and spurred further reliance on drugs and alcohol, as described by one participant following a sexual assault:

*I was like a confused person. It reached a point that I was drinking alcohol too much to the extent which on crossing the road I had no time to look if cars are coming or not to wait for them before crossing it. I was so tired with life, so getting an accident and die was all that I wanted.* -WH24.

Additionally, exposure to physical and sexual violence while using drugs also made some women particularly vulnerable to HIV acquisition, as described by a participant:

*Sometimes you can get a customer and he says, ‘I love you. Let’s go somewhere and have sex.’ Now you go thinking he is alone and when you arrive you find a group of people and they do to you whatever they want.* -WH11.

### HIV Acquisition, diagnosis and management

Being diagnosed with HIV was a traumatic event for women in the study: “*That was the worst day of my life, the day that I was told I am HIV positive”. –* WH29. Many participants were diagnosed with HIV when pregnant or when delivering; often engaging with the health system either for the first time ever or after a lapsed time. For some this coincided with death or illness of the partner due to undisclosed HIV infection, a traumatic and low point for women, as described by one participant following the death of her husband:

*We buried him and I came back home. And during that time, I was pregnant and the pregnancy was just big. My advice was that let me just go to the hospital and test [for HIV] and know how many months is my pregnancy. But my soul had already started shrinking. I did not expect it. Maybe he died because of the hernia, or the liver failing, or the kidney failing; all my thoughts is that my husband might have died because of the HIV infection.* -WH05.

While the timing of diagnosis, initiation of, and engagement in HIV care varied across the life histories of participants, ongoing drug use was a significant barrier to sustained HIV management. Some participants described that ART and the associated counseling messages, like the need for a healthy diet, were incompatible with using drugs, so they chose drug use over ART. Other participants discussed denial over their diagnosis and the decision to not engage with HIV care and continue drug use, as described by a participant:

*When I left that place [the clinic] I never followed up and I left [and continued] taking drugs [heroin]. That’s why I don’t even know what was the plan [to initiate ART], I know not of my CD4, I don’t know anything and I get weak every day and I get sick every day. -*WH10.

For a few participants, engagement in HIV care was a key turning point event as it included taking some control over their health, as described by a participant:

*I am back at home, then at home the money was somehow not enough, so I was no longer thinking of drugs of abuse. I sat down and asked myself, should I smoke, should I play a joke with the medications [ART], putting into consideration that I have the obligation of these grandchildren, moreover I am a matured adult now. So, I decided to reduce to start reducing the dose of smoking the drugs of abuse, I didn’t want to go on smoking. So that I can deal with the medication [ART] effectively.* -WH09.

The desire to protect a new or unborn child was for some a catalyst for managing their HIV, as described by a participant:

*If I do not use it [ART] I will get sick. When I get sick I will have to lie in bed, right? Who will take care of my child? I have to make every effort so that my child can live. And since she is breastfeeding I have to be [take ART] in time. I have to take the medicine so that she does not get the infection.* -WH02.

For some participants, pregnancy or a newborn was also a turning point to stop or decrease drug use, even if just for a brief period of time.

## Discussion

In this qualitative study, we found common events and themes across the life histories of WWUDHIV that influenced the start of drug use: death of a family member(s) or separation from family in adolescence/youth, migration to cities due to loss of family or social support, involvement in sex work, and intimate partners. Times in the life history where women were actively engaged in drug use were marked by trauma, poor mental health, and physical and sexual violence. Many participants noted HIV diagnosis as shocking, and for some, a traumatic event, with acquisition attributed to sexual violence, sex work, or partners. Initiating and/or continuing on ART was in direct conflict with drug use, with participants noting it was a choice between drug use and ART. Further, while a number of participants said they were diagnosed with HIV during pregnancy or following childbirth, their views on whether this led to the uptake or sustained use of ART were mixed. The accumulation of trauma over the life course affected women’s drug use trajectories and affected not only their vulnerability to HIV but also their mental health.

Catalysts for drug use found across the life histories of WWUDHIV in this study offer important areas for potential intervention to mitigate initiation of drug use and HIV acquisition. Our study found parental death to be a significant event due to both loss of economic and social support which impacted the drug use trajectories of many WWUDHIV. This is in line with data from other studies that have found orphanhood to be associated with drug use [28]. A life course perspective complements the life history approach by considering the influence of broader social, cultural, and economic factors that shape adolescent girls and young women’s life trajectories. Persistent conditions, such as ongoing engagement in sex work or poor mental health, can be examined within the context of women’s life course, providing insight into how these factors interact with cumulative trauma and influence vulnerability to HIV and substance use. By examining the accumulation of traumatic events and their influence on high, turning, or low points in women’s life trajectories, researchers and organizations that develop and implement public health programs can identify patterns and gain a deeper understanding of how trauma may contribute to drug use and vulnerability to HIV. Life course focused intervention approaches, which are aimed at optimizing health trajectories and address multiple levels of health determinants [29–31], could be a model to explore for drug use and HIV prevention (including provision of pre-exposure prophylaxis), particularly among at-risk female children or adolescents (e.g., those recently orphaned). Though high quality evidence is limited, interventions that take a life course approach have been shown to positively impact long-term outcomes including physical and mental health, and substance use [32–34]. Relevant to our findings where most women described orphanhood and discontinued school engagement, there is evidence that maintaining consistent school enrollment among children and orphaned adolescents, including the delivery of grants and the use of incentives for extended family who become caretakers, can be an effective approach to help them thrive in the life course [35]. Additionally, provision of reproductive and sexual health education and services to at-risk adolescents and young women, including contraception, health education, services for partner and sexual violence has been shown as an effective approach at preventing early pregnancy and intimate partner violence, which were common among participants in our study [35].

Participants in our study described how migration to Dar es Salaam, often in adolescence or young adulthood, influenced their initiation of drug use. Although evidence is scant, rural-to-urban migration is not necessarily associated with increased likelihood of drug use [36, 37]. However, most of the women in our study left their homes in particularly vulnerable situations due to loss of economic, familial, or social support. This is in contrast to many rural-to-urban migrants in the Africa region who frequently move back and forth between their home and the urban areas where they work, and thus benefit from and maintain familial and social support this way [38]. Our findings demonstrate that adolescent and young women who migrate are likely in particularly challenging situations and attention to these migrants and their needs is warranted.

Participants had a variety of descriptions of engagement with HIV services; a number saying they were diagnosed with HIV during or just after pregnancy. Prevention of mother-to-child HIV transmission (PMTCT) programs have dramatically reduced vertically acquired HIV in Africa [39], but our findings show that there may remain an important gap and opportunity for further intervention. Reasons for limited engagement with the health system among women who use drugs are likely multifaceted. People who use drugs are less likely to engage in the health system overall [40, 41], with anticipated or past experiences of drug use stigma being a reason for avoidance [42, 43]. Additionally, with engagement in sex work, standard clinic hours can be prohibitive to attendance [10, 44–46]. Nevertheless, most women in our study did engage with the health system in some capacity at different time points. Individualized case management approaches have shown promise in particularly vulnerable groups for supporting ART adherence, including among pregnant and/or post-partum women [47–49]. In addition, peer outreach has been used as a successful tool to increase engagement with HIV testing and care among hard to reach individuals such as sex workers and may offer promise among WWUDHIV [50]. While WWUDHIV represent a small proportion of the population in countries like Tanzania, as work to meet 95-95-95 goals continues [51], specialized approaches to support the needs of this group may be warranted.

Almost all participants in this study engaged in sex work to support drug use, with many citing no other opportunities for income generation for WWUD. Existing research shows that women who engage in sex work are at risk for a number of poor health outcomes, including physical and sexual violence, worsened mental health, HIV acquisition and onward transmission, and challenges with ART adherence [52–55]. For WWUDHIV these outcomes may be even more pronounced given their additional vulnerability due to increased lack of access to economic and social support [56, 57]. Among this particular population, where sex work is the primary way to sustain drug use, a combination of harm reduction approaches that can acknowledge and factor in the combination of gender-based vulnerability, poverty, and cumulative trauma may be critical to meeting the needs of this vulnerable population.

Poor mental health resulting from loss, sexual and physical violence, and other traumatic experiences was common among WWUDHIV in this study. The relationship between sexual violence and depression and/or post-traumatic stress disorder is well established in the literature [58], and engagement in sex work has an established link to experiences of sexual violence [59]. Trauma-informed psychosocial interventions for this population will be critical to address the health needs of WWUDHIV in this setting and have high exposure to trauma and violence. Trauma-informed cognitive behavioral therapy interventions have been used to reduce the harmful mental health impacts of sexual violence in other settings [60], have been used effectively among people living with HIV [60, 61], and could be adapted for WWUDHIV. Additionally, community-based links to effective substance use disorder treatment, including medications for opioid use disorder with integrated mental health services, which are increasingly available and accessible in Tanzania, may help to address the high burden of mental health problems in this population.

This study had a number of strengths. The use of a life history approach allowed participants to recall and relate their lives and life stories in way that provided information that may not have emerged in a typical semi-structured qualitative interview. Charting the life histories strengthened our ability to look across the narratives provided by participants and pull out themes and commonalities that might not have been as apparent using only standard qualitative coding approaches. Further, this approach allows for the identification of the potentially most impactful times in the life course to intervene on the trajectories of this particularly vulnerable population. Additionally, all interviews were formally coded, further strengthening the rigor of our analysis. This study was not without limitations. While the life history and life storytelling approach is a strength, we did not ask about specific events by name such as trauma, violence, or poor mental health, and so some of these elements of participant’s life histories may have been omitted depending on comfort to share or interest in recalling these events. Interviews also did not specifically ask for dates that significant events occurred, sometimes limiting the ability to pinpoint the timing of certain events in relation to others. However, we did find that overwhelmingly participants provided detailed and often linear stories about critical events across their lives. Additionally, we were limited to participants we were able to identify and recruit for the study. Given the multitude of factors that impact WWUDHIV’s lives and their marginalized place in Tanzanian society, it is possible our sample only represents one particular sub-section of this group. Future research should aim to include a more diverse range of participants to better understand the various factors that influence the life trajectories of WWUDHIV and inform targeted interventions.

## Conclusion

Findings from this study emphasize the importance of implementing interventions such as life course-focused approaches or trauma-informed psychosocial programming at various stages throughout the lives of women who use or are at risk of initiating drug use. Understanding timepoints of risk for substance use and HIV infection across the life course can allow for tailored interventions that target adolescent girls and young women with a focus on addressing trauma and mental health. Supporting and allocating resources to girls and women who are exposed to and/or impacted by trauma can help foster resilience and promote healthier coping mechanisms, which may ultimately reduce the likelihood of them turning to substance use as a means of coping. Furthermore, enhancing provider initiated testing and counseling for women at heightened risk of HIV acquisition and PMTCT programming and/or creating stronger linkage to ART and adherence programming for vulnerable women can significantly improve health outcomes for both women and their children. Ensuring interventions and programs are accessible and sensitive to the unique needs of WWUD can help to overcome barriers to care and improve engagement in HIV prevention and treatment services.

## Data Availability

There is no data held in a public repository. To protect the anonymity of participants, data may be made available upon reasonable request.

## Abbreviations

ART: antiretroviral therapy
HIV: human immunodeficiency virus
IDI: in-depth interview
PMTCT: prevention of mother to child transmission
PWUD: people who use drugs
WWUD: women who use drugs
WWUDHIV: women who use drugs living with HIV
